# Effects of ultrasound irradiation on Au nanoparticles deposition on carbon-coated LiNi_0.5_Mn_1.5_O_4_ and its performance as a cathode material for Li ion batteries

**DOI:** 10.1016/j.ultsonch.2021.105879

**Published:** 2021-12-16

**Authors:** Yasuyuki Tanaka, Hirokazu Okawa, Takahiro Kato, Katsuyasu Sugawara

**Affiliations:** Graduate School of Engineering Science, Akita University, 1-1 Tegatagakuen-machi, Akita, Akita 010-8502, Japan

**Keywords:** Sonochemical synthesis, Au nanoparticles, LiNi_0.5_Mn_1.5_O_4_, Carbon coating, Li-ion battery, AC, Alternating current, CS, Carbon and sulfur, CV, Cyclic voltammetry, SHE, Standard hydrogen electrode, TEM, Transmission electron microscopy

## Abstract

LiNi_0.5_Mn_1.5_O_4_ (LMNO) has attracted considerable attention as a Li-ion battery cathode material, owing to its high discharge voltage of 4.7 V (vs. Li/Li^+^) and high energy density. However, the electronic conductivity of LMNO is low, resulting in a low discharge capacity at high current density. To overcome this limitation, we deposited Au nanoparticles (NPs), which have a high conductivity and chemical stability at high battery voltages, on carbon-coated LMNO (LMNO/C) using ultrasound irradiation. Consequently, Au NPs that are ∼16 nm in size were deposited on LMNO/C, and ultrasound irradiation was reported to disperse the NPs on LMNO/C more effectively than stirring. Furthermore, the deposition of Au NPs on LMNO/C using ultrasound irradiation improved its electronic conductivity, which is related to an increase in the discharge capacity due to the reduction of Ni^4+^ to Ni^2+^ in LMNO/C at a high current density.

## Highlights

• The effect of ultrasound and LMNO on reduction of Au^3+^ to Au NPs are determined.
• Au NPs are successfully deposited on a 5-V-class cathode material using ultrasound.
• Ultrasound is superior to stirring for dispersing Au NPs on LMNO/C.
• Au NP deposition using ultrasound improves the electronic conductivity of LMNO/C.

## Graphical abstract

**Figure:**
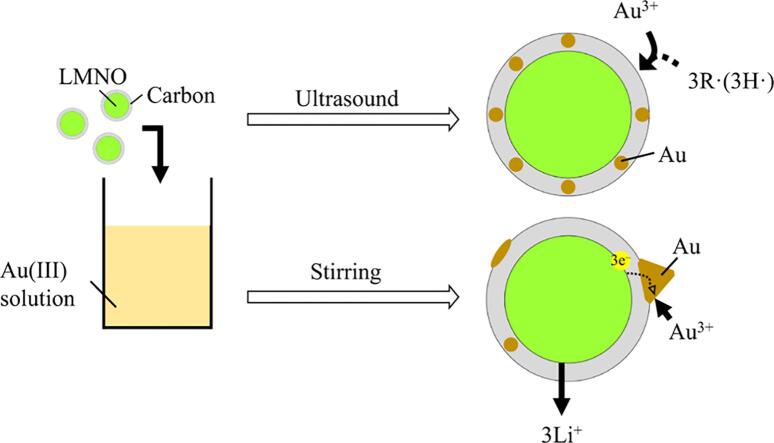

## 1 Introduction

Li ion batteries, which have high energy densities and energy capacities, are used as power supplies in electronic vehicles [1]. LiCoO_2_, a typical Li-ion battery cathode material, shows a discharge voltage of 3.8 V (vs. Li/Li^+^) and a discharge capacity of 148 mAh/g. Therefore, as a cathode material, the energy density of LiCoO_2_ is 562 Wh/kg [2]. The increase in the energy density of a battery requires increasing the discharge capacity per weight of cathode material and its discharge voltage. The discharge potentials of spinel LiMn_1.5_Ni_0.5_O_4_ (LMNO), olivine LiM_t_PO_4_, and orthorhombic Li_2_M_t_PO_4_F (where M_t_ = Co, Ni) are higher than that of layered LiCoO_2_ [3], [4], [5]. Accordingly, our research on cathode material has focused on LMNO, which shows excellent cycle performance and thermal stability [6]. LMNO shows high redox potentials of ∼ 4.7 V vs. Li/Li^+^, which comprises Ni^4+/3+^ and Ni^3+/2+^ (half-cell reaction stoichiometries of discharge are determined by the following Equations (1) and (2)), and discharge capacity calculated from Equations (1) and (2) is 135 mAh/g [7], [8].

(1) LiNi_0.5_(II)Mn_1.5_(IV)O_4_ → Li_0.5_Ni_0.5_(III)Mn_1.5_(IV)O_4_ + 0.5Li^+^ + 0.5e^−^

(2) Li_0.5_Ni_0.5_(III)Mn_1.5_(IV)O_4_ → Ni_0.5_(IV)Mn_1.5_(IV)O_4_ + 0.5Li^+^ + 0.5e^−^

The energy density of LMNO is estimated to be 650 Wh/kg, thus making it a promising candidate as an alternative to LiCoO_2_.

The crystal structure of spinel LMNO comprises NiO_6_ and MnO_6_ octahedral sites [9]; moreover, it crystallizes in Fd–3m and P4_3_32 space groups [10]. LMNO, with the P4_3_32 space group, can be obtained by the calcination of appropriate precursors under oxygen [11]; however, the Fd–3m form is obtained under air. Cathode materials that can be synthesized in air are extremely useful in an industrial prospective because it facilitates scaling up of synthesis amount of LMNO. Interestingly, the cycle performance of the Fd–3m form is higher than that of the P4_3_32 form [12]. However, the electronic conductivities of both forms (∼1.0 × 10^-^^7^ S/cm) are lower than that of LiCoO_2_ [13], [14], leading to poorer discharge capacities at high current densities.

Multiple studies that address this limitation have involved coating the surface of LMNO with conductive carbon and/or doping it with other metal ions. For example, when some Ni in LMNO is substituted with Co and Fe, thus forming LiNi_0.4_Co_0.1_Mn_1.5_O_4_ and LiNi_0.4_Fe_0.1_Mn_1.5_O_4_. Furthermore, the electronic conductivity and cycle performance are improved because binding energy of Co–O and Fe–O is stronger than that of Mn–O, thus suppressing the structural disintegration that occurs because of the Jahn–Teller distortion during Li insertion–desertion. However, this method causes a comparative decrease in Ni content, which reduces the discharge capacity contribution of the Ni^2+/3+^ and Ni^3+/4+^ redox pair [15].

In a related study, the LMNO surface was coated with conducting carbon via the thermal decomposition of sucrose, glucose, C_2_H_2_ gas, and xerogel synthesized using resorcinol and formaldehyde [16], [17], [18], [23]. Although carbon coating was reported to improve electronic conductivity, an excess amount of carbon disrupted Li-ion diffusion to and from the LMNO because excessive carbon coating causes the aggregation of LMNO particles and the generation of a thick carbon layer on LMNO, which increases Li^+^ diffusion distance between the LMNO and electrolyte [17]. Moreover, for the carbon coating process, carbon can reduce Mn^4+^ in LMNO to Mn^3+^ by reaction with oxygen. LMNO also shows discharge capacity contribution from the Mn^4+/3+^ couple. However, the redox potential of Mn^4+/3+^ is ∼4.0 V vs. Li/Li^+^, which is lower than that of Ni^4+/3+^ and Ni^3+/2+^. Furthermore, during the charging process, Mn^3+^ in LMNO causes disproportionation of Mn^2+^ and Mn^4+^ and Mn^2+^ dissolves in the electrolyte [19]. To summarize, for LMNO, a thin coating of an extremely small amount of carbon (∼1 wt%) is optimal [16], [17].

LMNO may decompose the electrolyte during charging at low current densities because the potential becomes extremely high (4.8–4.9 V vs. Li/Li^+^). Both oxide and Au coatings on active cathode materials have been explored as a means to overcome this limitation, e.g., a 2 wt% Bi_2_O_3_ layer on LiMn_1.42_Ni_0.42_Co_0.16_O_4_, which has a potential similar to that of LMNO, has been demonstrated to suppress electrolyte decomposition; however, during the discharge process, Bi_2_O_3_ coating was reduced to Bi [20]. Furthermore, coating LMNO with Au to a thickness of 25 nm has been demonstrated to suppress the reaction between LMNO and electrolyte. However, during discharge and charge processes, Au coating disrupted Li^+^ intercalation and deintercalation [21].

In our study, we examined the deposition of Au nanoparticles (NPs) on carbon-coated LMNO (LMNO/C). The deposition of dispersed Au NPs on the carbon layer of LMNO/C can improve its electronic conductivity without negatively affecting Li diffusion because the electronic conductivity of Au is higher than that of carbon. Moreover, the redox potential of the Au^0/3+^ redox pair is +1.52 V vs. a standard hydrogen electrode (SHE). During charging, this high redox potential suppresses the dissolution of deposited Au NPs via the oxidation of Au to Au^3+^. Au may be expected to suppress the reaction between the LMNO and electrolyte.

The use of ultrasound in the synthesis of active materials has been reported with its effect being largely dependent on its frequency [22]. For instance, Sivakumar et al. reported that nano-sized LMNO particles synthesized using a sol–gel method involving ultrasound irradiation at 20 kHz demonstrated good electrochemical cyclability [23]; Kim et al. reported that high-purity LiCoO_2_ can be quickly synthesized at relatively low temperatures using ultrasound at 20 kHz [24]; González et al. reported that a graphene/amorphous FeOOH composite synthesized using ultrasound at 20 kHz was generated as fine particles with the excellent dispersion of two phases and that it exhibited a higher charge/discharge capacity than that of graphite [25]. Furthermore, Masjedi-Arani et al. reported that Zn_2_SiO_4_ NPs synthesized using ultrasound irradiation at 20 kHz were more homogeneous and well-dispersed than samples synthesized using sol–gel and hydrothermal methods and former method exhibited superior electrochemical activity [26].

In the aforementioned studies, low-frequency ultrasound was used to produce mechanical effects to promote dispersion and synthesize fine particles. However, the synthesis of active materials using high-frequency ultrasound to produce chemical effects has been reported. For instance, Okawa et al. reported that amorphous FePO_4_·2H_2_O was successfully synthesized as fine (sub-micron) and homogeneous particles via the oxidation of Fe^2+^ to Fe^3+^ using oxidative radicals generated by ultrasound at 200 kHz without H_2_O_2_ [27]. Furthermore, to produce metal NPs, polyol, sputtering, and sonochemical methods have been reported [28], [29], [30], and it has been reported that Pd, Au, Ag, and Pt NPs can be synthesized using reductants generated by ultrasound irradiation [31], [32], [33], [34]. For example, Yoshida et al. reported that Au NPs generated from Au^3+^ by ultrasound irradiation can be deposited on the surface of acetylene black and carbon-coated LiFePO_4_ (LFP/C) [35], while Saliman et al. reported that Pd NPs deposited on LFP/C to 0.5 wt% using ultrasound improved its discharge capacity at 10C [34]. However, the discharge voltages of LFP/C used in these studies were 3.4 V (vs. Li/Li^+^) and, to our knowledge, there are no studies about the detailed explanation of ultrasound for depositing metal NPs on the surface of a 5-V-class cathode material.

Accordingly, in this study, we aimed to improve Ni^4+/3+^ and Ni^3+/2+^ discharge capacity of LMNO/C at high current densities by depositing well-dispersed Au NPs on its surface using ultrasound. We examined the effects of ultrasound irradiation on the size and morphology of Au NPs deposited on LMNO/C and evaluated the battery performance of Au NPs deposited LMNO/C (LMNO/C·Au) as a cathode material.

## 2 Experimental

### 2.1 Au NPs deposition on LMNO/C using ultrasound irradiation

LMNO was synthesized using a sol–gel method. A 100-mL reaction solution of ion-exchanged water containing 0.0225 mol of Mn(CH_3_COO)_2_·4H_2_O (99.0%, Wako), 0.0075 mol of Ni(CH_3_COO)_2_·4H_2_O (99.0%, Wako), and 0.0157 mol of LiOH·H_2_O (98.0%, Wako) was prepared. The reaction solution was stirred at 500 rpm for 1 h at 90 °C, followed by the elimination of excess water at 90 °C without stirring. The mixture was dried under vacuum at 55 °C for 24 h, and the resultant solid was calcined at 400 °C for 10 h under atmospheric conditions after crushing. Underatmospheric conditions, the calcined sample was ground by ball milling for 1 h and then calcined at 800 °C for 10 h.

Sucrose was used as a carbon source to coat the surface of LMNO particles. The thermal decomposition temperatures of sucrose with and without LMNO were examined using thermogravimetric–differential thermal analysis (TG–DTA; Rigaku TG8120) in air. A mixture of LMNO and sucrose was heated at 190 °C for 30 min in air, followed by a temperature increase from 190 to 280 °C at a heating rate of 2 °C/min in air. When the temperature reached 280 °C, the sample was removed from the electric furnace. The amount of carbon coating on the surface of LMNO was estimated using a carbon and sulfur (CS) analyzer (EMIA-220 V; HORIBA).

Au NPs were deposited on LMNO/C by the following procedure: LMNO/C (0.1 g) was suspended in 2-propanol solution (1.4 mM, 45 mL) prepared from 2-propanol (99.9%, Wako) and ion-exchanged water. The air dissolved in the prepared solution was purged by bubbling Ar at 100 mL/min for 30 min. Then, 5 mL of Au^3+^ solution (1 mM) prepared using HAu(III)Cl_4_ (99.9%, Wako) was added to the solution, which was then irradiated by ultrasound for 20 min under Ar atmosphere. The output and frequency of ultrasound were set to 200 W and 200 kHz, respectively. The ultrasonic power reached to the solution (50 mL) was estimated to be 14.5 W using a previously reported calorimetry method [36]. Using a water bath, the temperature of the solution during ultrasound irradiation was maintained in the range of 20–25 °C. The sample obtained from the solution was collected after being thoroughly washed using ion-exchanged water.

The crystal structures of LMNO/C and LMNO/C·Au were determined using powder X-ray diffraction (RINT 2200; Rigaku) with CuKα radiation between 10° and 90° at a scanning rate of 2°/min. The accelerating voltage and applied current were 40 kV and 40 mA, respectively. The morphology of Au NPs deposited on LMNO was observed using transmission electron microscopy (TEM). The amount of Au NPs deposited on LMNO/C was determined from the change in the Au ion concentration of the reaction solution before and after ultrasound irradiation or stirring. Aqua regia was added in the irradiated solution to dissolve Au NPs and obtain Au ions. The Au ion concentration was measured using an inductively coupled plasma (ICP) spectrophotometer (SPS5510 SSI; Hitachi).

### 2.2 Electrochemical performance of LMNO/C·Au

The cathode material was prepared by mixing the active material, acetylene black as a conductive agent, and polyvinylidene fluoride (PVDF) as a binder at a weight ratio of 8:1:1 using a pestle and mortar. The cathode material was then made into a paste with *N*-methyl pyrrolidone and applied to Al foil to a thickness of 100 μm using a blade. The cathode material was then dried at 55 °C under vacuum. A 1-cm^2^ round section of the film was cut and used as the cathode in a battery. Galvanic cells were then assembled in a glove box saturated with Ar gas. Li (1 cm^2^) was used as the anode, 1 M LiPF_6_ in ethylene carbonate/dimethyl carbonate (1:1, *v/v*) was used as the electrolyte, and a 25-μm monolayer of polypropylene film (Celgard #2500; Celgard) was used as the separator. Battery tests were performed with constant current rates of 0.5, 1.0, 2.0, 5.0, and 10C in the voltage range of 3.5–4.9 V vs. Li/Li^+^ at 20 °C using a battery test system (PXF2011; Kikusui). Cyclic voltammetry (CV) measurements were performed with a sweeping speed of 10–120 mV/min using an automatic polarization system (HSV-110; Hokuto). The charge transfer resistance of the cathode was determined from the Nyquist plots of alternating current (AC) impedance measurements obtained using a chemical impedance analyzer (IM3590; HIOKI) in the frequency range from 0.10 Hz to 100.00 kHz.

## 3 Results and discussion

### 3.1 Au NPs deposition on LMNO/C using ultrasound irradiation

**Fig. 1:**
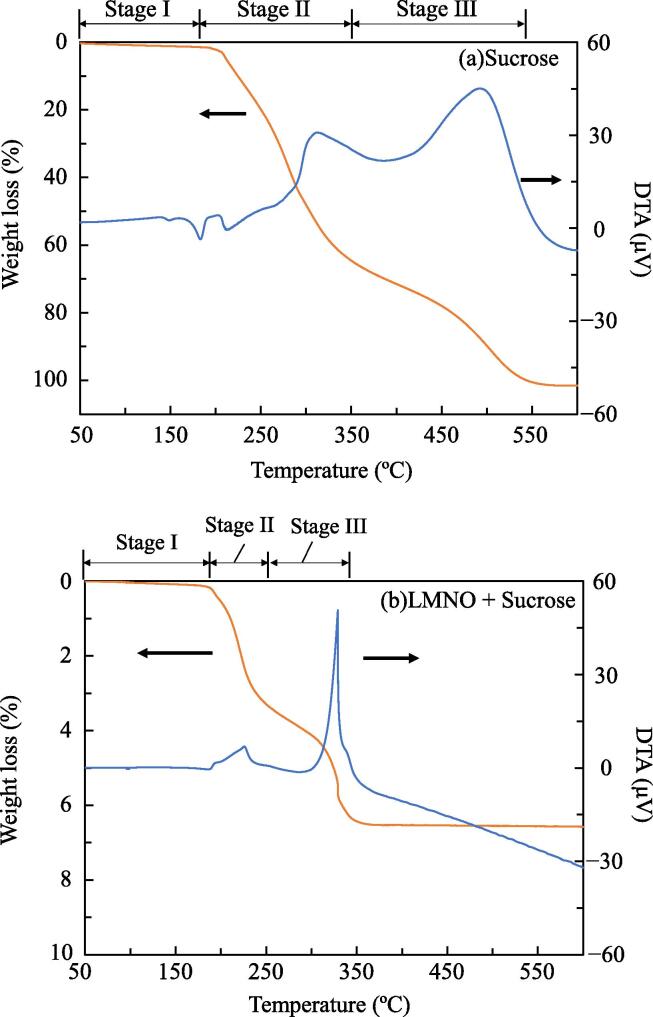
TG and DTA curves of (a) sucrose and (b) mixture (sucrose (6.3 wt% of mixture) and LMNO) with a heating rate of 5 °C/min in air.

The LMNO effect on the thermal decomposition of sucrose was examined and optimum temperature for carbon coating of LMNO was determined. Fig. 1(a) shows the TG and DTA curves for sucrose in air. The TG curve shows three distinguished weight-loss stages: stage I: 50–183 °C; stage II: 183–350 °C; and stage III: 350–543 °C. Stage I is the evaporation of surface-adsorbed water, and the mass loss is 1.5 wt% at 183 °C. Stage II is the evaporation of water generated by the thermal decomposition of sucrose, and the mass loss is 64.6 wt% at 350 °C. Sucrose is decomposed to water and carbon [37], and the stage-II reaction is determined by the following equation:

(3) C_12_H_22_O_11_ → 11H_2_O + 12C

The theoretical weight loss by H_2_O release for this process is 58%. Stage III is the combustion of carbon generated from sucrose, as indicated by its exothermic nature, and the mass loss is 33.9 wt% at 543 °C.

Figure 1(b) shows the TG and DTA curves of a mixed sample comprising sucrose and LMNO recorded in air. The sucrose content of the sample is 6.3 wt%; the TG curve of the mixture is similar to that of sucrose and shows three-stage weight loss: stage I: 50–187 °C; stage II: 187–252 °C; and stage III: 252–342 °C. However, the mixture shows the exothermic reaction at a lower temperature than that observed for sucrose without LMNO. This is most possibly owing to LMNO acting as a catalyst, which promotes the oxidation of carbon to CO_2_ [38]. Therefore, we set the calcination temperature at 280 °C, which is the temperature below the start of combustion of carbon coating (∼40 wt% of the added sucrose remains as carbon).

**Fig. 2:**
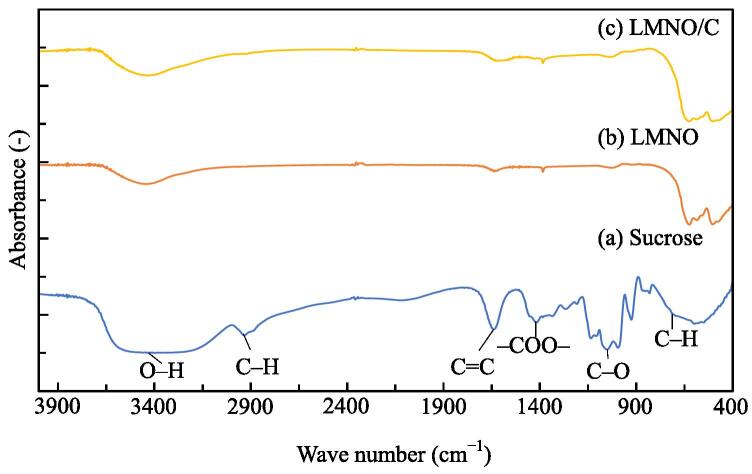
FTIR spectra of (a) sucrose, (b) LMNO, and (c) LMNO/C (C: 1.26 wt%).

Figure 2 shows the Fourier transform infrared spectroscopy (FTIR) spectra of sucrose, LMNO, and LMNO/C. Peaks at 780, 1025, 1407, 1671, 2925, and 3370 cm^−1^ are observed for sucrose and are attributed to the C–H stretching vibration, C–O bending vibration, –COO– symmetric vibration, C=C stretching vibration, C–H stretching vibration, and O–H stretching vibration, respectively [39]. The peaks for LMNO/C (C: 1.26 wt%) are similar to those for LMNO; this result shows that carbon on LMNO does not remain as sucrose.

The diffraction peaks of LMNO (Fig. S1(a)) show that LMNO crystallizes in the Fd–3m space group[40]. The XRD pattern shows a peak close to 2θ = 43.7°, which is attributed to a small amount of NiO impurity [41]. When synthesizing LMNO, a lack of oxygen can result in NiO generation [42], which often appears when LMNO is synthesized using the sol–gel method. In the next step, LMNO and sucrose were mixed and heated in an electric furnace. When the temperature reached 280 °C, the sample was immediately taken out of the oven to prevent the oxidation of carbon (Fig. S1(b)). As determined using a CS analyzer, the carbon content of LMNO/C was 1.26 wt%. The primary particle size of LMNO/C was estimated to be 212 ± 59 nm using TEM. Finally, using ultrasound, Au NPs were deposited on LMNO/C (C: 1.30 wt%). The Au content of LMNO/C·Au (Fig. S1(c)) is 0.11 wt%; no Au peak was observed because the amount of Au is extremely low.

The XRD patterns of LMNO/C and LMNO/C·Au are similar that of LMNO, which indicates that the LMNO structure is maintained throughout carbon coating by heating and deposition of Au NP using ultrasound irradiation. LMNO/C·Au was synthesized by adding LMNO/C to a Au^3+^ solution, followed by ultrasound irradiation. Ultrasound irradiation to the solution generates hydrogen radicals (H·) and hydroxyl radicals (OH·) (Equation 4) [43]. 2-Propanol (R–H) acts as a OH· scavenger, and the organic radicals (R·) reduce Au^3+^ to Au [44].

(4) H_2_O → H· + OH·

(5) R-H + OH· → R· + H_2_O

(6) R-H + H· → R· + H_2_

(7) Au^3+^ + 3H· → Au^0^ + 3H^+^

(8) Au^3+^ + 3R· → Au^0^ + 3R’ + 3H^+^

Au NPs were synthesized by the reduction of Au^3+^ using H· and R· generated by ultrasound (Equations 7 and 8). Therefore, the rapid reduction of Au^3+^ using reduction radicals generated using ultrasound is necessary to synthesize Au NPs.

The valency of Mn in LMNO with the Fd–3m space group is primarily reported as Mn^4+^, including a small amount of Mn^3+^. Fig. S2 shows the first charge–discharge curve of LMNO. The charge capacity between 3.8 and 4.3 V (vs. Li/Li^+^) is attributed to Mn^3+/4+^. This charge curve shows that some of the Mn in LMNO exists as Mn^3+^. The charge capacity for Mn^3+/4+^ is 10.9 mAh/g in the first cycle, which corresponds to 7.4% of the total charge capacity. The redox potential of Mn^3+/4+^ in LMNO is ∼4.0 V vs. Li/Li^+^, which is equivalent to 1.0 V vs. SHE. Mn^3+/4+^ in LMNO can reduce Au^3+^ to Au metal because the redox potential of Au^0/3+^ is 1.5 V vs. SHE. However, Ni^2+/3+^ and Ni^3+/4+^ in LMNO cannot reduce Au^3+^ because the redox potential of Ni^2+/3+^ and Ni^3+/4+^ is higher than that of Au^0/3+^. Therefore, electrons released from LMNO by the oxidation of Mn^3+^ to Mn^4+^ can directly reduce Au^3+^ to Au^0^ or via the carbon of LMNO/C. Moreover, Li^+^ is released from the LMNO (deintercalation) in the solution when Mn^3+^ in LMNO is oxidized to Mn^4+^. Thus, to clarify the effect of LMNO on the reduction of Au^3+^, the Li and Mn concentrations in the solution after the deposition of Au NPs on the LMNO/C using ultrasound irradiation or stirring (500 rpm) were determined. The Mn and Li concentrations released from LMNO/C (C: 0.91 wt%) using ultrasound irradiation are 3.5 ppm and 1.1 ppm, corresponding to 0.4 wt% and 2.0 wt% of the initial amounts of Mn and Li in LMNO/C. However, the Mn and Li concentrations in LMNO/C (C: 0.91 wt%) after stirring are 0.3 ppm and 1.3 ppm, corresponding to 0.0 wt% and 2.5 wt% of the initial amounts of Mn and Li in LMNO/C. Thus, the Mn concentration released from LMNO under ultrasound irradiation is higher than that under stirring. This result is attributed to the fact that ultrasound irradiation promotes the dissolution of LMNO prepared using HAuCl_4_ in acidic conditions (pH 3). After the dissolution of LMNO, Li is released in the solution, increasing its concentration in the solution. However, after stirring, the Li concentration from LMNO is higher than that after ultrasound irradiation, although there is a low Mn concentration. Thus, Au NPs are primarily deposited on LMNO/C by radicals under ultrasound irradiation and by Mn^3+^ in LMNO with stirring.

**Fig. 3:**
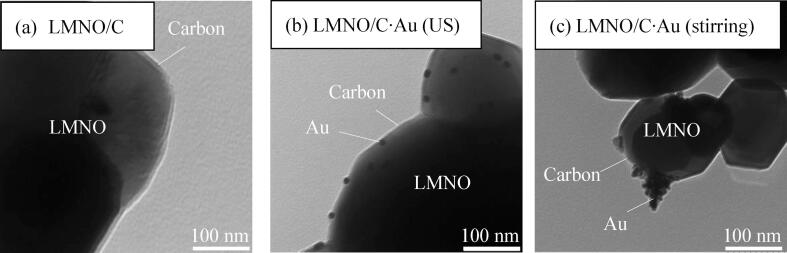
TEM images of (a) LMNO/C (C: 1.30 wt%), (b) LMNO/C·Au prepared using ultrasound (C: 1.30 wt%, Au: 0.11 wt%), and (c) LMNO/C·Au prepared using stirring (C: 0.91 wt%, Au: 0.78 wt%).

Figure 3 shows the TEM images of LMNO/C and LMNO/C·Au prepared using ultrasound irradiation and stirring. Fig. 3(a) shows the amount of carbon in LMNO/C is 1.30 wt% and the carbon layer is 5 nm thick. Fig. 3(b) shows LMNO/C·Au prepared by ultrasound irradiation, and the amounts of carbon and Au are 1.30 wt% and 0.11 wt%, respectively. Au NPs are well dispersed on the carbon layer of LMNO/C as globular particles having a size of ∼16 nm. Fig. 3(c) shows Au deposited on LMNO/C (C: 0.91 wt%) using stirring, and the amount of Au is 0.78 wt%. The Au yields deposited on LMNO/C from Au^3+^ with stirring and ultrasound irradiation were 81% and 11%, respectively. However, Au NPs, having sizes of 12–60 nm, deposited on LMNO/C using stirring are nonuniform and exhibit particle growth. The area of Au deposited on LMNO/C has a higher electronic conductivity than that of carbon coated on LMNO. Consequently, electrons released from LMNO react with Au^3+^ via Au NPs on LMNO/C, which leads to particle growth (similar to dendrite). This dendrite-like Au is unfavorable for batteries because it can form short circuits. The Au yield deposited on LMNO/C using ultrasound irradiation is lower than that of Au deposited on LMNO/C using stirring, which is considered to be the effect of microjets from cavitation on Au deposition. The cavitation effect can possibly prevent the deposition of Au NPs generated far from the LMNO/C surface using ultrasound irradiation. The Au yield seems to be low because only Au NPs generated on (or near) the surface of the LMNO/C are deposited. Under naked-eye observation, the filtrate of the reaction solution after ultrasound irradiation was violet. Thus, a considerable amount of Au NPs remained in the solution; however, using ultrasound for dispersing Au NPs on the LMNO/C is important.

### 3.2 Electrochemical performance of LMNO/C·Au

**Fig. 4:**
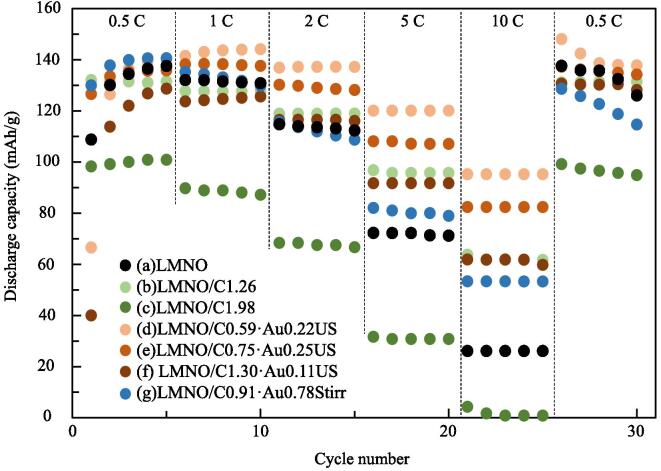
Rate performances of (a) LMNO, LMNO/C ((b) C: 1.26 wt% and (c) C: 1.98 wt%) and LMNO/C·Au ((d) C: 0.59 wt%, Au: 0.22 wt% (ultrasound), (e) C: 0.75 wt%, Au: 0.25 wt% (ultrasound), (f) C: 1.30 wt%, Au: 0.11 wt% (ultrasound), and (g) C: 0.91 wt%, Au: 0.78 wt% (stirring)) measured by changing current value from 0.5 to 10C each 5 cycles.

Figure 4 shows the rate performances of LMNO, LMNO/C, and LMNO/C·Au as cathode materials from 0.5 to 10C, followed by cycling at 0.5C between 3.5 and 4.9 V (vs. Li/Li^+^). Carbon-coated amount and Au-deposited amount on LMNO were summmarized in Table 1. The discharge capacity of LMNO synthesized in this study (Fig. 4(a)) was similar to that of LMNO reported in previous study (70 mAh/g) at 5C [45]. The discharge capacities of two samples of LNMO were measured to confirm experimental error in this study (Fig. S3). Higher one in two samples is shown in Fig. 4. Moreover, owing to carbon coating on the LMNO surface, the discharge capacity improved from 26.0 to 61.7 mAh/g at 10C. Finally, we deposited Au NPs on LMNO/C using ultrasound irradiation to improve the discharge capacity of LMNO/C at 10C, and the discharge capacities of LMNO/C·Au (C: 0.59 wt%, Au: 0.22 wt% (Fig. 4(d)) and C: 0.75 wt%, Au: 0.25 wt% (Fig. 4(e))) were improved 1.4–1.5-fold compared to that of LMNO/C (C: 1.26 wt% (Fig. 4(b)) at 10C. Although some of Li and Mn from LMNO gets dissolved under ultrasound irradiation, these dissolutions do not affect charge and discharge cycling. However, the discharge capacities of LMNO/C·Au (C: 0.91 wt%, Au: 0.78 wt%) prepared using stirring demonstrates no improvement in discharge capacity compared to LMNO/C. Au NPs deposited on LMNO/C using stirring grow without dispersion, thus agglomerating and having no effect on the electronic conductivity of LMNO/C. Thus, at high current density, the improved dispersal for Au deposition improves the electronic conductivity and discharge capacity of LMNO/C.

**Table 1:** Carbon-coated amount and Au-deposited amount on LMNO using each condition.

Condition	Carbon coated amount (wt%)	Au deposited amount (wt%)	Treatment type of Au deposition	Abbreviation
(a)	0	0	–	LMNO
(b)	1.26	0	–	LMNO/C1.26
(c)	1.98	0	–	LMNO/C1.98
(d)	0.59	0.22	Ultrasound	LMNO/C0.59·Au0.22US
(e)	0.75	0.25	Ultrasound	LMNO/C0.75·Au0.25US
(f)	1.31	0.11	Ultrasound	LMNO/C1.31·Au0.11US
(g)	0.91	0.78	Stirring	LMNO/C0.91·Au0.78Stirr

**Fig. 5:**
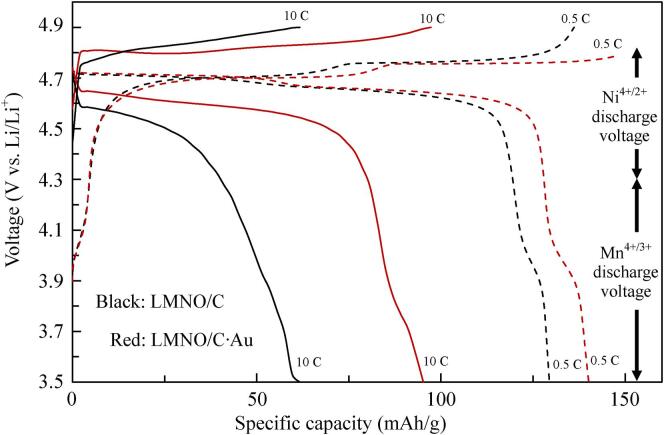
Charge and discharge curves for LMNO/C (C: 1.26 wt%) and LMNO/C·Au prepared using ultrasound (C: 0.59 wt%, Au: 0.22 wt%) at 0.5C (5th cycle) and 10C (25th cycle).

Figure 5 shows the charge–discharge curves of LMNO/C (C: 1.26 wt%) and LMNO/C·Au (C: 0.59 wt%, Au: 0.22 wt%) treated by ultrasound at 0.5C for each fifth cycle. The charge–discharge curves of LMNO/C at 0.5C are similar to those of LMNO/C·Au. The discharge curves of LMNO/C and LMNO/C·Au are divided into two regions: 4.8–4.3 V vs. Li/Li^+^ from reduction of Ni^4+^ to Ni^2+^ and 4.3–3.5 V vs. Li/Li^+^ from Mn^4+^ to Mn^3+^. The total discharge capacity of LMNO/C is 129.5 mAh/g based on the contributions of 119.5 mAh/g from Ni^4+^ to Ni^2+^ (92%) and 10.0 mAh/g from Mn^4+^ to Mn^3+^ (8%). The total discharge capacity of LMNO/C·Au is 140.2 mAh/g based on the contributions of 127.9 mAh/g from Ni^4+^ to Ni^2+^ (91%) and 12.3 mAh/g from Mn^4+^ to Mn^3+^ (9%). Thus, the deposition of Au NPs by ultrasound irradiation improves the discharge capacity of Ni^4+/3+^, Ni^3+/2+^ and Mn^4+/3+^. Moreover, LMNO/C·Au demonstrates a higher discharge capacity at the high current density of 10C compared to that of LMNO/C. This result shows that the resistance of the battery cell decreases by the deposition of Au NPs, improving its charge–discharge performance.

**Fig. 6:**
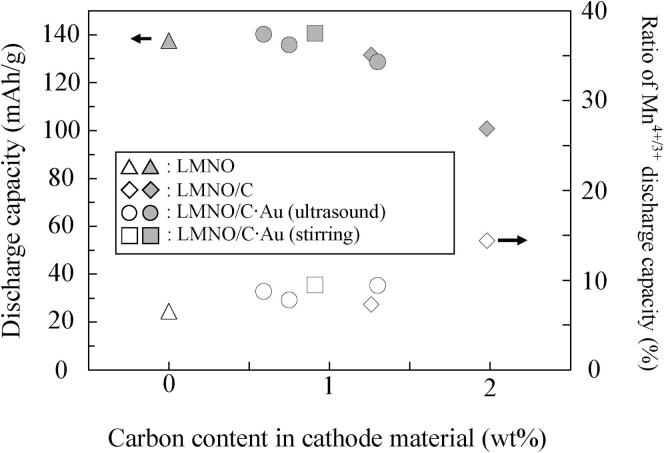
Changes in discharge capacity and the contribution of Mn^4+/3+^ discharge capacity (4.3–3.5 V vs. Li/Li^+^, 0.5C) per total discharge capacity (4.8–3.5 V vs. Li/Li^+^, 0.5C) for LMNO, LMNO/C, and LMNO/C·Au owing to carbon content.

Figure 6 shows the effects of carbon coating and the deposition of Au NP on the total discharge capacity and contribution of Mn^4+/3+^ per total discharge capacity at the fifth cycle for the low current density of 0.5C. Triangles, diamonds, circles, and squares denote LMNO, LMNO/C, LMNO/C·Au prepared using ultrasound irradiation, and LMNO/C·Au prepared using stirring, respectively. Gray and white fillings show total discharge capacity (4.8–3.5 V vs. Li/Li^+^) and ratio of Mn^4+/3+^ discharge capacity (4.3–3.5 V vs. Li/Li^+^). The ratio of Mn^4+/3+^ discharge capacity is defined using the following formula:

(9) $$\begin{array}{c} Ratio\;of\;Mn^{4+/3+}\;discharge\;capacity\;\left(\%\right)\\ \;=\frac{{\mathrm{Mn}}^{4+/3+}\;discharge\;capacity\;\left(mAh/g\right)}{\mathrm{Total}\;\mathrm{discharge}\;\mathrm{capacity}\;(mAh/g)}\times 100 \end{array}$$

The ratio of Mn^4+/3+^ for the discharge capacity of LMNO/C (C: 1.98 wt%) is higher than those of LMNO and LMNO/C (C: 1.26 wt%) because Mn^4+^ in LMNO/C (C: 1.98 wt%) is reduced to Mn^3+^ by the carbon coating process. Oxygen in LMNO reacts with carbon generated from sucrose during the carbon coating process, which results in the reduction of Mn^4+^ to Mn^3+^ in LMNO by loss of oxygen [46]. The charge valency of Mn does not change after charge–discharge cycles because the oxygen lost from LMNO by carbon coating is not recovered during charge and discharge processes. Therefore, the reduction of Mn^4+^ by carbon coating seems to increase Mn^4+/3+^ discharge capacity and decrease Ni^4+/3+^ and Ni^3+/2+^ discharge capacities at 4.7 V vs. Li/Li^+^.

**Fig. 7:**
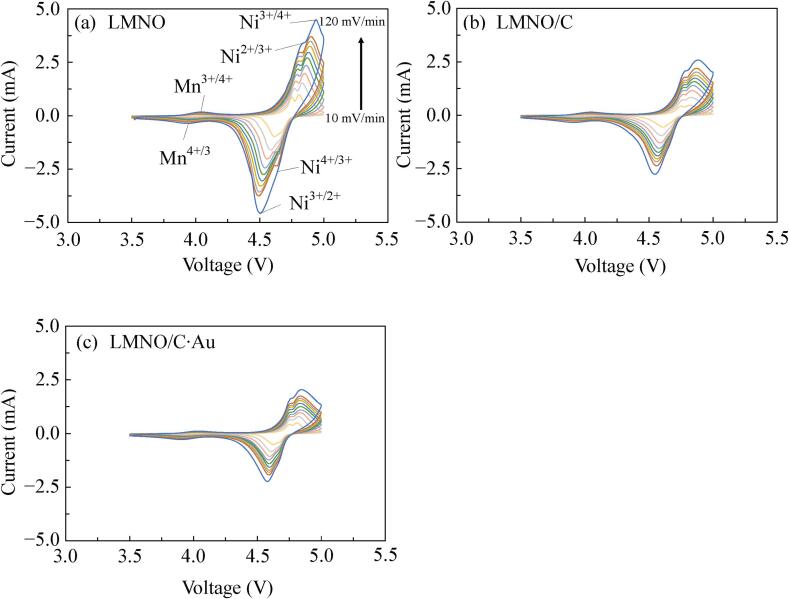
CV results for (a) LMNO, (b) LMNO/C (C:1.03 wt%), and (c) LMNO/C·Au (C: 0.75 wt%, Au: 0.25 wt%) at sweeping rates from 10 to 120 mV/min.

Figure 7 shows the CV profiles of LMNO, LMNO/C (C: 1.03 wt%), and LMNO/C·Au (C: 0.75 wt%, Au: 0.25 wt%) prepared using ultrasound to examine the surface reaction of the cathode electrode. CV tests were conducted using samples after five charge–discharge cycles at 0.5C. Measurements were performed over a voltage range of 3.5–5.0 V vs. Li/Li^+^ with sweeping rates of 10–120 mV/min. The three anodic peaks for LMNO around 4.0, 4.7, and 4.8 V at 10 mV/min correspond to Mn^3+/4+^, Ni^2+/3+^, and Ni^3+/4+^. The three cathodic peaks for LMNO around 3.9, 4.6, and 4.7 V at 10 mV/min correspond to Mn^4+/3+^, Ni^3+/2+^, and Ni^4+/3+^. These peaks are observed for LMNO/C and LMNO/C·Au without peaks come from the decomposition of electrolytes. As the sweeping rate increases, the polarization peak of LMNO becomes significant because LMNO with its low electronic conductivity cannot respond to a high sweeping rate. The CV curves for LMNO/C·Au are similar to those of LMNO and LMNO/C.

**Fig. 8:**
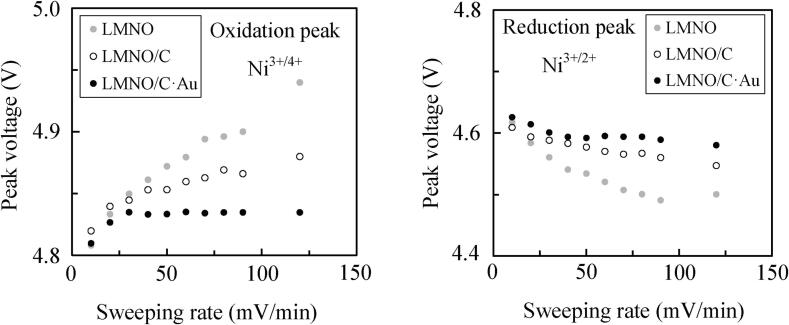
Plots of the oxidation and reduction peak voltages of LMNO, LMNO/C (C:1.03 wt%), and LMNO/C·Au (C: 0.75 wt%, Au: 0.25 wt%) at each sweeping rate from 10 to 120 mV/min.

However, the peak voltages of three samples are different at each sweeping rate. Figure 8 shows the anodic peak voltages for Ni^3+/4+^ and cathodic peak voltages for Ni^3+/2+^ in LMNO, LMNO/C, and LMNO/C·Au as revealed by CV curves (Fig. 7) for each sweeping rate. At all sweeping rates, the anodic peak voltage for Ni^3+/4+^ in LMNO/C·Au is the least, and the cathodic peak voltage for Ni^3+/2+^ in LMNO/C·Au is the highest of the three samples. This result shows that the electron transfer at the surface of LMNO/C·Au is the fastest of these three samples. Thus, Au NP deposition on LMNO/C using ultrasound irradiation improves electronic conductivity.

**Fig. 9:**
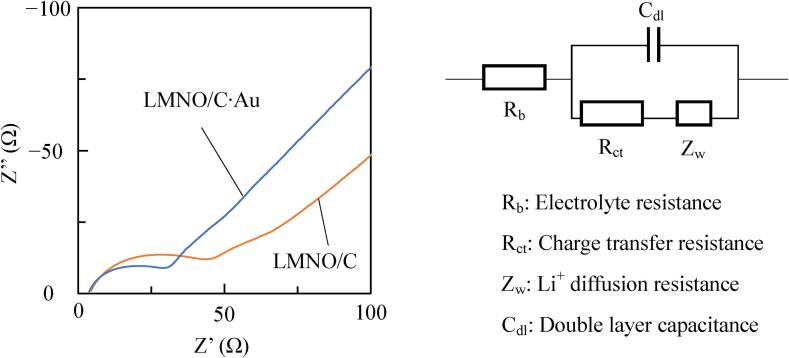
Nyquist plots for LMNO/C (C:1.26 wt%) and LMNO/C·Au (C: 0.75 wt%, Au: 0.25 wt%).

Figure 9 shows the equivalent circuit model proposed from the Nyquist plots for LMNO/C (C: 1.26 wt%) and LMNO/C·Au (C: 0.75 wt%, Au: 0.25 wt%) prepared using ultrasound after five charge–discharge cycles at 0.5C. AC impedance measurement was performed from 0.1 Hz to 100 kHz. In this equivalent circuit, R_b_ is the electrolyte resistance, R_ct_ is the charge transfer resistance of the cathode, Z_w_ is the Warburg impedance, and C_dl_ is the double-layer capacitance of the cathode. The battery voltages of LMNO/C and LMNO/C·Au in AC impedance measurement are 3.84 V and 3.74 V vs. Li/Li^+^, respectively. The charge transfer resistances of LMNO/C and LMNO/C·Au are 43 Ω and 29 Ω, respectively, demonstrating that the deposition of Au NPs improves the charge transfer resistance of LMNO/C. Thus, increase in the discharge capacity of LMNO/C after the deposition of Au NPs using ultrasound irradiation is attributed to the improvement of charge transfer resistance.

## 4 Conclusion

We demonstrated that the discharge capacity of LMNO/C at high current density is successfully improved by the deposition of Au NPs using ultrasound irradiation, which allows the formation of well-dispersed NPs of ∼ 16 nm in size on LMNO/C. However, Au particles deposited on LMNO/C using stirring show particle growth because Au^3+^ reacts with electrons, which are released from LMNO, via Au NPs on LMNO/C. Furthermore, the reduction of Au^3+^ to Au NPs using ultrasound irradiation suppresses the reduction of Au^3+^ by LMNO and inhibits the agglomeration of Au NPs. Note that the deposition of Au NPs on LMNO/C using ultrasound successfully improves the Ni^4+/2+^ discharge capacity of LMNO/C at 10C. The discharge capacity of LMNO/C·Au (C: 0.59 wt%, Au: 0.22 wt%)) prepared using ultrasound is improved ∼ 1.5 times compared to that of LMNO/C (C: 1.26 wt%) at 10C and does not promote the electrolyte decomposition. The charge transfer resistance of LMNO/C·Au is lower than that of LMNO/C. These results show that the deposition of Au NPs improves the electronic transfer rate of LMNO/C, leading to less voltage drop at a high discharge current density. LMNO/C·Au (C: 0.59 wt%, Au: 0.22 wt%) demonstrated good performance; furthermore, the role of Au for battery performance was understood through this study. However, there are two challenges: i) to suppress the dissolution of LMNO controlling the pH at which Au NPs are deposited because ultrasound irradiation causes LMNO dissolution under acidic conditions, and ii) to increase the yield of NPs using high-frequency ultrasound, such as 600 kHz, with a reduced mechanical effect.

Recently, for all-solid-state batteries, an Au thin film placed at the electrolyte interface improves the reversibility of Li metal anode utilization [47]. Therefore, the deposition of Au NPs using the proposed ultrasound method may be applied not only for Li ion batteries with electrolyte solutions but also for all-solid-state batteries. In future, we aim to identify alternative materials for Au NPs because Au is expensive to use as a cathode material of Li-ion batteries with electrolyte solutions.

## CRediT authorship contribution statement

**Yasuyuki Tanaka:** Writing – original draft. **Hirokazu Okawa:** Conceptualization, Funding acquisition, Writing – original draft. **Takahiro Kato:** Writing – review & editing. **Katsuyasu Sugawara:** Writing – review & editing.

## Declaration of Competing Interest

The authors declare that they have no known competing financial interests or personal relationships that could have appeared to influence the work reported in this paper.
