# Supplementary material for: Effects of ultrasound irradiation on Au nanoparticles deposition on carbon-coated LiNi0.5Mn1.5O4 and its performance as a cathode material for Li ion batteries
Source: Ultrason Sonochem. 2021 Dec 16;82:105879. doi: 10.1016/j.ultsonch.2021.105879 (PMC8799617; doi:10.1016/j.ultsonch.2021.105879)
Supplement: Supplementary data 1 [file mmc1.docx]

Supplemental file

553

NiO

(c) LMNO/C∙Au

(b) LMNO/C

Intensity (a.u.)

(a) LMNO

111

311

222

400

331

511

440

531

444

551

10

30

50

70

2θ (deg.)

90

LMNO (Fdm, PDF No. 01-080-2162)

622

Fig. S1 XRD patterns of (a) LMNO, (b) LMNO/C (C: 1.26 wt%), and (c) LMNO/C·Au prepared using ultrasound (C: 1.30 wt%, Au: 0.11 wt%)

4.8

4.6

4.4

4.2

4.0

3.8

3.6

3.4

3.2

3.0

Voltage (V vs. Li/Li^+^)

0

50

100

150

Specific capacity (mAh/g)

Fig. S2 First charge–discharge curves of LMNO at 0.5 C

The first charge capacity of Mn^3+/4+^

0.5 C

1 C

2 C

5 C

10 C

0.5 C

Fig. S3 Rate performances of LMNO samples measured by varying current value from 0.5 to 10 C after each 5 cycles (Error bars represent standard deviation, n = 2 (p ≤ 0.01).)

Cycle number

0

10

20

30

160

140

120

100

80

60

40

20

0

Discharge capacity (mAh/g)
